# Knowledge and Knowledge Needs about Lyme Disease among Occupational and Recreational Users of the Outdoors

**DOI:** 10.3390/ijerph17010355

**Published:** 2020-01-05

**Authors:** Sarah E. St. Pierre, Odette N. Gould, Vett Lloyd

**Affiliations:** 1 Department of Psychology, Mount Allison University, 49A York St., Sackville NB E4L 1C7, Canada; sestpierre@mta.ca; 2 Department of Biology, Mount Allison University, 49A York St., Sackville NB E4L 1C7, Canada; vlloyd@mta.ca

**Keywords:** Lyme Disease, Lyme disease knowledge, Lyme disease prevention, health prevention education

## Abstract

As the prevalence of Lyme disease increases across Canada, it is imperative that the educational needs of at-risk groups be identified. The current study compared the level of knowledge and the knowledge needs about Lyme disease among individuals that spend time outdoors for work and for recreational purposes. Between December 2018 and February 2019, a survey was distributed to outdoor organizations across New Brunswick, Canada. Within the current sample of 137 individuals, 36% spent time outdoors for their occupation and 64% for recreational activities. Results showed no significant difference between these groups with regard to their level of knowledge, perceived efficacy and performance of various methods of prevention, and educational needs. Overall, the entire sample reported a low level of knowledge about Lyme disease. Participants perceived each prevention behavior to be at least somewhat effective, and behaviors perceived to be more effective were more likely to be carried out, but the performance of the behaviors varied. The most frequently performed behaviors included wearing long pants and protective footwear. Participants identified several aspects of Lyme disease about which they would like to have more information. The findings call attention to the specific needs of at-risk groups that must be considered when developing educational interventions.

## 1. Introduction

Lyme disease is the most common vector-borne disease in North America [1,2]. As a result of climate change and other factors, endemic areas for Lyme disease continue to expand in the eastern and central provinces of Canada, including southern New Brunswick [3,4]. The immature nymphal stage, as well as the adult stage, of the tick vector, *Ixodes scapularis*, is a small ectoparasite that may easily go unnoticed when attached to people. This, coupled with non-specific symptoms of infection, can result in missed or incorrect diagnoses [4]. It has been found that 12.3% of ticks in New Brunswick are infected with *Borrelia burgdorferi*, the most common Lyme disease-causing bacterial species in North America [5]. The proportion of people becoming ill from tick bites varies based on exposure (number of tick bites), duration of tick feeding, identification and treatment of tick bites and/or early disease, regional differences in *B. burgdorferi* genotypes as well as less understood aspects of human physiology [5,6,7]. Wilhemsson et al. estimate that 8% or more individuals bitten by an infected tick develop a *Borrelia* infection [7]. Due to increased exposure to ticks, the Canadian Lyme Disease Foundation and public health agencies have identified individuals that spend time outdoors for work as well as hikers, horse riders, hunters, sport fishers and other recreational users of the outdoors as being at a higher risk of becoming infected with Lyme disease [3,8]. In order to effectively prevent the transmission of Lyme disease, employees and recreational users of the outdoors should practice tick encounter prevention. The use of protective clothing, bug repellents, showering after being outdoors, checking their body for ticks, avoiding tick-infested areas at work sites, and using tweezers to remove a tick when an attached tick is found have all been proposed [9,10]. However, little research has explored whether these prevention behaviors are perceived as effective or feasible by the people most at risk of being exposed to infected ticks.

While research efforts in Canada have not yet addressed the unique risk that Lyme disease presents to at-risk groups, several studies carried out elsewhere have examined the serological prevalence of *Borrelia burgdorferi* sensu lato, the agent that causes Lyme disease, as well as gaps in knowledge about Lyme disease among foresters and agricultural workers [10,11,12,13,14]. According to the Central Register of Research Occupational Diseases in Poland, the prevalence of serological response to *B. burgdorferi* in farmers increased from 17% to 76% between 2000 and 2014 [14]. Furthermore, infectious and parasitic diseases were shown to account for 62% of all occupational diseases among Polish farmers, with tick-borne diseases being the most frequent at 93% [14]. In France, rates of infection in workers in high-risk professions have been shown to vary between 13% and 22% [12] An important factor that leads to the use of protective measures against Lyme disease, such as wearing protective clothing and checking the body for ticks, is the belief that one is at risk of being infected [12,13,15,16]. Many studies find that those who work in at-risk professions report adopting safety measures [10,11,12], although many of these workers also report feeling that they know too little about Lyme disease [11].

Research has been conducted within North America, examining existing knowledge of ticks and tick encounter prevention behaviors in the general public. Bayles et al. [17] found that the majority of their study participants reported an awareness of ticks and their ability to spread Lyme disease. Participants who visited recreational parks within suburban areas were more likely to be “not at all” concerned with tick-borne diseases than individuals in exurban or rural parks. The most frequently reported safety behaviors were walking in the center of the trail, checking the body for ticks, avoiding wooded or grassy areas and using a bug repellent spray, and the least commonly reported preventative behaviors included wearing long pants or a long-sleeved shirt and tucking pants into socks [17].

In a similar study, Butler et al. [18] carried out a survey in a high-risk area and showed that the most commonly reported safety behavior adopted was checking one’s body for ticks (68%) and the least frequent was the use of insect repellent (38%). These authors also showed that participants perceived each safety behavior as being effective at reducing the risk of tick-borne diseases and perceived efficacy was highly positively correlated with the performance of each behavior [18]. Similarly, in a web-based survey in a region of Quebec that was newly at risk for Lyme disease, it was found that only 54% of the respondents had heard of Lyme disease before filling out the survey, and that of these, less than half used protective measures that they perceived as being highly effective [19]. In contrast, Hallman et al. [20] found that all participants visiting three recreational parks in a high-risk area of New Jersey were aware of Lyme disease and most knew of the serious consequences, yet 60% of the participants did not take any precautions. In this sample, however, knowing someone with Lyme disease and the belief that the disease is difficult to cure was positively related to the performance of safety behaviors.

There have been some efforts to reduce the prevalence of Lyme disease and other tick-borne diseases across Canada by promoting behaviors reducing tick encounters. For example, the Public Health Agency of Canada launched a social marketing campaign in 2014 to increase awareness of safety behaviors against Lyme disease in both Canadians participating in outdoor activities and health professionals [16]. A survey [16] showed that public awareness of Lyme disease had increased, particularly in provinces with low entomologic risk. In 2015, the Public Health Agency of Canada released a *Federal Framework on Lyme Disease in Canada* [21] that called attention to the importance of public education as well as collaboration with stakeholders, including patients, their advocates, and health care providers. Review of this framework was proposed for 2020 [21].

The current study explored the extant knowledge and educational needs regarding Lyme disease among individuals who use the outdoors for their occupation as well as those who spend time in the environment for recreational purposes. In consideration of the limited access that recreational users of the outdoors may have to safety training and information, we hypothesized that individuals who spend time in the environment for recreational purposes would have gaps in knowledge that are not apparent in individuals who spend time outdoors for work. A secondary goal was to identify the safety behaviors more likely to be adopted and the educational needs identified by members of both groups.

## 2. Materials and Methods

Participants were recruited from professional and recreational organizations across New Brunswick (NB) via email. Of the 19 organizations initially approached, 11 agreed to send the link for the online survey to their members, including the Agricultural Alliance of NB, NB Federation of Small Woodlot Owners, National Farmers’ Union NB, Hammond River Angling Association, NB Wildlife Federation, NB Equestrian Association, Dieppe Fly-Tying Club, Tantramar Seniors’ College, Tantramar Outdoors Club, Les Amis de la Nature du Sud-est Inc., and Club d’Ornithologie du Madawaska. The recruitment email also offered pre-paid postage envelopes containing a paper version of the survey upon request. Among the organizations, two requested paper versions of the survey for their members. In total, we received three responses on paper and 134 online submissions. Of these 137 individuals (46 females, 89 males, 2 other), ages ranged from 21 to 80 years old (M = 55, SD = 14), with 120 having completed post-secondary education and 16 having completed high school (one participant omitted answering this question).

The survey included 48 questions, took approximately 20 min to complete and was available on paper or online via LimeSurvey. The survey is presented in the Supplementary Materials. The survey was designed by the study authors and based on survey items used in previous research [11,19]. The survey was translated into French by a professional translator and made available in both languages to all respondents. The first section of the survey consisted of demographic questions including age, gender, education, outdoor activities, county, community (suburban, urban, or rural), and experience with Lyme disease. In analyses, suburban and urban respondents were combined to create two comparison groups, namely urban and rural. Respondents from counties in the northern part of the province (Madawaska, Restigouche, Gloucester, Northumberland, York, Victoria, Kent, and Carleton) were compared to respondents in the southern part of the province (Sunbury, Queens, Saint John, Kings, Charlotte, Albert and Westmorland counties) as the Lyme disease risk is considered higher in the south than the north of the province [22]. The second section of the survey contained questions about the frequency at which participants performed various safety behaviors on a 7-point scale ranging from never to always with the midpoint labeled as “sometimes” (e.g., “How often do you wear long pants to avoid ticks infected with Lyme disease”). In the third section, participants were asked to rate how effective they believed these preventive behaviors to be on a 7-point scale ranging from not at all effective to very effective, with the mid-point in the scale labeled as neutral (e.g., “How effective do you think wearing long pants is in avoiding ticks infected with Lyme disease?”). The fourth section of the survey addressed participants’ history of exposure to tick bites, and knowledge of the disease, including (a) a self-rating of knowledge on 7-point scale ranging from (1) not at all, to 7 (extremely) knowledgeable, and (b) an open-ended question asking for a description of the symptoms of Lyme disease occurring 1 to 4 weeks after infection in an open-ended question. The fifth section consisted of questions regarding the participants’ knowledge about symptoms and best treatments for Lyme disease (e.g., “In your opinion, what is the best treatment for Lyme disease?” and “Where have you learned about ticks and Lyme disease?” For these questions, multiple choice answers were provided, and a blank space for supplementary choices was provided. The sixth section of the survey addressed participants’ knowledge needs by providing a list of topics (e.g., “Where do I get treatment for Lyme disease?”) and for each asking how important this topic is on a 7-point scale ranging from “not at all important for me to learn more about this” to “very important for me to learn more about this.” Lastly, the survey provided open-ended questions for additional comments about Lyme disease education such as “What are other aspects of Lyme disease that you would like more information about?”

## 3. Results

### 3.1. Statistical Analyses

Descriptive statistics (means, standard deviations and percentages) were used to summarize demographic data. For testing hypotheses comparing groups (i.e., occupation and location groups), chi square tests were used for all nominal variables, and a t-test for independent samples with Cohen’s d as a measure of effect size was used to compare the group means on the self-rating of knowledge variable. For group comparisons on participation in safety behaviors and perceived effectiveness of safety behaviors, independent groups t-tests were carried out on the means of the self-ratings, and Holm’s sequential Bonferroni was used to control for family-wise error. The final set of analyses presented results for the entire sample combined. For (a) participation in safety behaviors, (b) perceived effectiveness of safety behaviors, and (c) the rated importance of different types of sought-after information about Lyme disease, the group mean for each variable was compared to the middle value on the 7-point scale using one sample t-tests. For each series, Holm’s sequential Bonferroni was used to control for family wise error. Pearson correlations were calculated between the frequency of carrying out safety behaviors and the perceived effectiveness of these behaviors. Finally, the first two authors worked together to sort the responses to open-ended questions into categories. All analyses were carried out using IBM SPSS Statistics for Windows, version 25 (IBM Corp, Armonk, NY, USA).

### 3.2. Demographics

The sample included 137 participants, with 64.2% (*n* = 88) of participants having spent time outdoors only for recreational purposes. Individuals who participated in both recreational and professional activities were categorized as being in the occupational group. The most commonly reported activities by participants in the recreational group were hiking (*n* = 58, 65.9%), sport fishing (*n* = 51, 57.9%) home gardening (*n* = 46, 52.3%), sport hunting (*n* = 35, 39.8%), dog ownership (*n* = 36, 40.9%), recreational paddling (*n* = 30, 34.1%), and golfing (*n* = 11, 12.5%). The participants who worked outdoors (*n* = 49, 35.8%), included individuals who owned a small wood lot (*n* = 31, 63.3%), farmers (*n* = 19, 38.8%), tree planters (*n* = 12, 25.5%), forestry professionals (*n* = 2, 4.2%) and a dog trainer (*n* = 1, 2.1%). Many individuals participated in more than one professional or recreational activity. Demographic information for the two groups is provided in Table 1. Chi square tests indicated that the Occupation group was slightly better educated (Χ^2^(1, *N* = 136) = 4.35, *p* = 0.03), and was more likely to contain rural residents (Χ^2^(1, *N* = 137) = 15.75, *p* < 0.001). The two groups were similar for other demographic variables. As can be seen in Table 1, several participants reported a history of tick bites. Within the current sample, only one participant reported having had a diagnosis of Lyme disease but 14 were unsure about whether they had been infected. Of these participants, two reported that their symptoms began six or more years prior to the completion of the survey and one participant indicated that they were still suffering at the time of the study. Only a small number of participants (*n* = 27) provided information about how they had removed a tick, with 74% using tweezers and 26% using their fingers.

### 3.3. Occupational and Recreational Users of the Outdoors

It was hypothesized that individuals who spend time in the environment for recreational purposes would be less knowledgeable about Lyme disease than people who spend time outdoors for work. To test this hypothesis, an independent samples t-test was carried out on ratings of self-reported level of knowledge showed that recreational (Mean (M) = 3.41, Standard deviation (SD) = 1.51) and occupational (M = 3.96, SD = 1.70) respondents did not differ significantly on this variable (t (133) = 1.92, *p* = 0.057, d = 0.34. Second, a series of independent samples t-tests with Holm’s sequential Bonferroni correction for familywise error were used to determine whether significant differences occurred between professional and non-professional users of the outdoors in regard to their level of safety participation and their perception of the efficacy of these safety measures. No significant differences were obtained. Each respondent was also asked whether they had received information about Lyme disease from a series of sources: work-based training, health care practitioners, public health, recreation association, news media, social media, and friends/family. The two groups differed significantly on only two of these sources: more occupation (20%) than recreation (6%) respondents had received work-based training (Χ^2^(1, *N* = 132) = 7.55, *p* = 0.006), and more recreation (48%) than occupation (25%) respondents had received information from friends and family (Χ^2^(1, *N* = 132) = 6.42, *p* = 0.01). Lastly, the two groups did not differ on self-reported educational needs.

### 3.4. Location

Follow up exploratory analyses were conducted to look at differences between individuals who reside in the northern and southern regions of the province and individuals who reported residence in a suburban/urban or rural community. These groups were compared on self-rated level of knowledge, frequency of safety behaviors, perceived effectiveness of safety behaviors, and educational needs using Holm’s sequential Bonferroni correction. No differences were obtained between the groups, except that respondents from the north of the province (M = 6.31, SD = 1.35) were more likely to want information about how to identify ticks than respondents from the south (M = 5.42, SD = 1.85, t (120) = 3.07, *p* = 0.003, d = 0.54).

### 3.5. Tick Bite Prevention Behaviors

**Figure 1:**
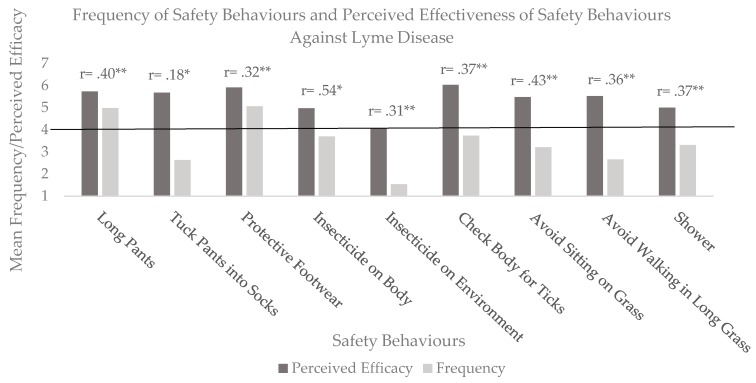
Mean ratings for frequency and perceived efficacy of safety behaviors. * *p* < 0.05, ** *p* < 0.01.

Given the lack of differences between the subgroups as described above, subsequent analyses were carried out on the entire sample. First, we assessed the frequency of performing Lyme disease prevention behaviors on a scale ranging from 1 (never) to 7 (always). These data are presented in Figure 1. We used one-sample t-tests with Holm’s Bonferroni correction to compare the frequency and perceived effectiveness of each safety behavior to the midpoint of 4 (sometimes).

Only two safety behaviors were used significantly more often than ‘sometimes’, namely the frequency of *wearing long pants* (t (130) = 5.80, *p* < 0.001, d = 0.50) and *protective footwear* t (129) = 5.85, *p* < 0.001, d = 0.51). In contrast, many safety behaviors were used significantly less than sometimes, namely, *tucking pants into socks* (t (131) = −8.27, *p* < 0.001, d = 0.71), *spraying insecticide on the environment* (t (130) = −20.65, *p* < 0.001, d = 1.81), *avoidance of sitting on grass* (t (129) = −434, *p* < 0.001, d = 0.38) and *avoidance of walking on long grass* (t (130) = −8.84, *p* < 0.001, d = 0.49). In terms of perceived effectiveness, except for *using insecticide on the environment* (t (114) =0.45, *p* = 0.65, d = 0.04), all other safety behaviors were rated significantly more effective than the neutral point on the scale (*p* < 0.001, d ranging from 0.65 to 1.69).

Bivariate correlations (shown in Figure 1 above each column) consistently showed significant and positive relationships between frequency and perceived efficacy of safety behaviors, indicating that these individuals were more likely to use safety behaviors they judge to be effective.

### 3.6. Lyme Disease Knowledge

**Figure 2:**
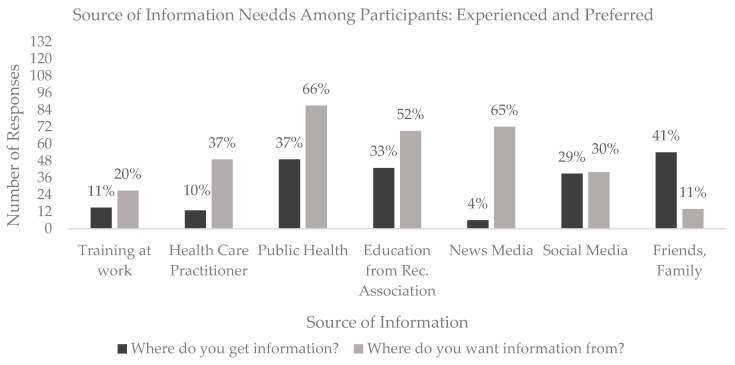
Where participants have accessed information regarding Lyme disease and where they would like to access information in the future.

A one-sample t-test showed that the self-rating of knowledge of Lyme disease (M = 3.61, SD = 1.59) was significantly lower than the midpoint on the 7-point scale (t (134) = −2.86, *p* = 0.005, d = 0.24) indicating a relatively low level of self-confidence in knowledge. The source of information from which participants have accessed information as well as where they would like to gather information about Lyme disease in the future is displayed in Figure 2. Overall, the current sample reported that the **most** frequent source of information was *friends and family* and *public health* sources and the sources **least** reported were *health care practitioners* and the *news media*. Respondents reported that they desired more information from *public health* and the *news media* in the future (some participants indicated having accessed and wanting access to information from more than one source).

**Figure 3:**
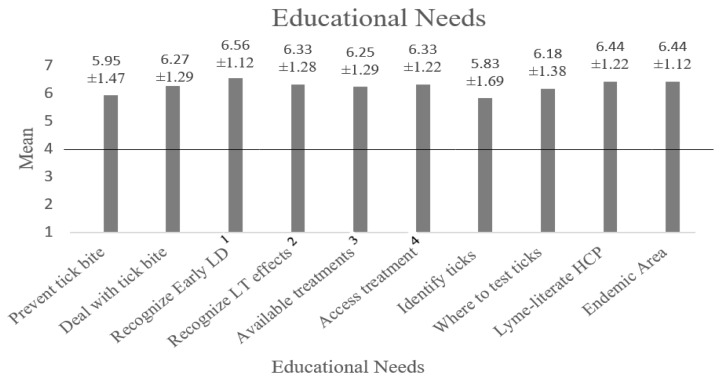
The self-reported educational needs of individuals that spend a lot of time outdoors. ^1^ How to recognize Lyme disease in the first days of infection. ^2^ How to recognize the long-term effects of Lyme disease. ^3^ Lyme-literate HCP = where to find health care providers who are knowledgeable about Lyme disease. ^4^ Endemic Area = how to find out if an area contains many ticks infected with Lyme disease.

To assess educational needs, participants were asked to rate how important various types of information related to Lyme disease were for them to learn more about on a scale that ranged from 1 (not at all important for me to learn more about this) to 7 (very important for me to learn more about this). Figure 3 shows the mean response for each type of information and provides the precise mean value and standard deviation above each column. The desire to access information was significantly higher than neutral for each area of knowledge, with *p* < 0.001 and Cohen’s d ranging from 0.69 to 2.29.

When asked to select the most effective treatment for Lyme disease, most participants chose antibiotics that are prescribed by a physician to be the best treatment (*n* = 86, 56.6%) followed by integrated care addressing many symptoms simultaneously (*n* = 62, 40.8%). Very few participants reported that a skin cream used to control the rash would be the most effective treatment for Lyme disease (*n* = 4, 2.6%), and none of the participants reported that the disease could resolve on its own. Participants were also asked to list the symptoms that they believed to occur one to four weeks following a bite from an infected tick. Only a subsample (*n* = 79) chose to answer this open-ended question. The symptoms that participants associated with Lyme disease in the earliest stage are listed in Table 2.

### 3.7. Additional Information Requested (n = 28)

A content analysis of responses to the question “What are other aspects of Lyme disease that you would like more information about?” revealed the following five themes. (a) At-risk Areas and Peak Season (*n* = 6). Several participants expressed interest in obtaining information about what areas in the region and what times of year presented a high-risk for becoming infected with Lyme disease. For example, one respondent stated “*Knowing which areas are active with Lyme-infected ticks would be very helpful. I feel like all the precautions are not worth the trouble if my area is not a hot-spot. I don’t know if some areas are more high risk than others or if the risk is the same across the province, or region.”* (b) Tick Identification, Removal and Post-Removal (*n* = 3). Some participants responded that they would like to be educated about how to recognize ticks that carry Lyme disease in Atlantic Canada, as well as what to do when a tick is removed from the skin. For example, one person wanted “*clear protocol explanations for how to remove ticks and if they should be brought with you to the doctor—how to proceed.”* (c) Symptoms and Treatment (*n* = 11). Frequent comments addressed wanting more information regarding the symptoms when one is infected with Lyme disease, including when treatment is delayed or not provided. Examples included *“Side effects, side effects from delayed/neglected treatment”; “Is there a time limit where the effects of Lyme are permanently irreversible?”* and *“Is it life threatening? “*Particularly striking in this category was confusion about the appropriate time to seek treatment. As one respondent noted “*How soon should an individual seek medical attention if feeling symptoms of LD? Given the symptoms are similar to other more minor illnesses, there is a sense of hesitation to (for lack of better words) to request antibiotics for Lyme if you have a headache, muscle pain and fever for instance.* (d) Pets (*n* = 3). A few participants expressed confusion about the treatment of pets infected with Lyme disease compared to the diagnostic protocol for humans: *“Why is it so much easier to get a diagnosis for animals than it is for humans?”* A separate concern was the possibility of pet to human transfer: “*Pets get lots of ticks. I’ve heard different information about whether cats/dogs can get Lyme disease, how it affects them (how you can tell they have it), whether they can transfer it to you,* etc. *Would be great to clear that up.”* (e) Canadian Health Care Systems (*n* = 6) Many comments addressed the perceived difficulty of obtaining a prompt and accurate diagnosis for Lyme disease in the Canadian health care system. For example, respondents wrote: *“How are physicians trained to deal with this serious disease?”; “Have any advancements been made in diagnosis and treatment?”;* and *“Do you still have to have testing and treatment done in the USA for best results?”*

## 4. Discussion

The current study aimed to compare the use and perceived efficacy of tick avoidance safety behaviors, and existing knowledge and educational needs of occupational and recreational users of wilderness areas. The results of the survey indicated that there were no significant differences between people who work in the wilderness and people who participate in recreational activities in the wilderness in terms of perceived level of knowledge, educational needs, frequency of use and perceived efficacy of safety behaviors. As would be expected, occupational respondents had received more work-based information about Lyme disease, although, surprisingly, this applied to only 20% of these workers. (Because of the small number of individuals involved, statistical comparisons between workers who had received workplace training and those who had not were inconclusive, although mean differences suggest that trained workers may have been more likely to adopt safety behaviors). Few differences were observed between individuals residing in the northern and southern parts of the province and individuals living in urban/suburban and rural areas. Given the rural nature of much of the province, the lack of differences between the urban and rural groups may not be surprising, although it does contrast to past findings [15]. What is more surprising and concerning is that safety behaviors are not adopted more frequently by individuals who work in the wilderness and those who live in areas considered Lyme disease risk areas (i.e., the southern part of the province). Some informational interventions have been carried out with both groups, but seemingly they are not successful at enhancing uptake of tick bite prevention behaviors.

In interpreting these results, it is important to note that the limited number of respondents in each category may have been a factor in explaining non-significant findings, though it should be noted that the consistently small effect sizes in the group comparisons argues that the groups were similar on the majority of the compared variables. A further limitation of the present study is the high educational levels in the sample. Most participants indicated that they had at least some post-secondary education (university, college or trade school), suggesting that individuals with less education may not have been able or willing to fill out the survey. Future research using a much larger sample and comparing preventive behaviors and Lyme disease knowledge in groups differing in educational attainment may be useful in addressing this issue.

Two main findings emerge from these data. The first is that behaviors linked to avoiding tick bites are being carried out relatively infrequently. In previous literature Butler et al. [18] reported checking the body for ticks as the most frequently performed safety behavior. Bayles et al. [17] found checking the body for ticks, walking in the center of the trail, avoiding grassy areas and using a bug repellent spray to be the most frequently performed behaviors among their participants. In contrast, in the present study none of these safety behaviors were carried out frequently. Indeed, only wearing long pants and protective footwear was done more frequently than “sometimes”. We did, however, support Butler et al.’s [18] finding in two ways. First, we found that the adoption of safety behaviors was correlated with their perceived efficacy, and, second, there was no obvious relationship between the level of burden of each behavior and its frequency of use. Indeed, our results suggest that protective behaviors are only frequently adopted if they protect the individual from immediate discomfort, such as long pants protecting one’s legs from scratches and protective footwear preventing foot and ankle injuries. More in-depth research using mixed methods may be useful in investigating when, why and how people use Lyme disease prevention methods. More data is also needed about the effectiveness of the behaviors purported to prevent Lyme disease infection.

A second finding from this study is the low levels of confidence individuals have in their knowledge about Lyme disease and the clear desire for more information. The self-reported level of knowledge about Lyme disease was significantly lower than the (neutral) midpoint on 7-point scale ranging from “very little knowledge” to “very high level of knowledge.” Moreover, for each area of knowledge, participants’ desire to access information was significantly higher than the neutral point on the scale. Participants indicated that it was very important for them to learn more about tick bite prevention, identification, testing and how to deal with tick bites. Presently, the most frequent sources of information about Lyme disease were friends/family, but these respondents stated that they wanted more information from public health and news media sources. This suggests that public health agencies need to play a larger role in providing the public with needed information. Moreover, the answers to our open-ended question concerning what information is needed also suggest that public education programs need to be more targeted and provide information about what specific areas of the province are most at risk, and how to cope after a tick bite, including recognizing symptoms, getting a tick tested for disease, and getting prompt and effective medical care.

## 5. Conclusions

In conclusion, our findings call attention to a desire for further information about all aspects of tick exposure and Lyme disease among individuals that spend time outdoors in New Brunswick. In line with previous research, it was evident that the perceived efficacy of safety behaviors does not necessarily lead to adoption of those behaviors. To develop effective public health interventions, future research must assess not only the effectiveness of each intervention but the causal determinants of the adoption of preventative behaviors. Lastly, the desire for increased knowledge of at-risk individuals should be used to guide the development of educational interventions in this province and potentially in other newly at-risk regions.

## Figures and Tables

**Table 1:** Demographic characteristics and tick exposure of the participants.

Characteristics	Groups	*n* = 137	Occupational 49 (35.8%)	Recreational 88 (64.2%)
Mean age		55.0	52.8	56.2
Post-secondary Education		120 (87.6%)	47 (95.9%)	73 (82.9%)
Gender	Men	89 (64.9%)	35 (71.4%)	54 (61.3%)
	Women	46 (33.6%)	13 (26.5%)	33 (37.5%)
Location	Urban/Suburban	73 (53.8%)	15 (30.6%)	58 (65.9%)
	Rural	64 (46.7%)	34 (69.4%)	30 (34.1%)
County	North	57 (41.6%)	18 (36.7%)	39 (44.3%)
	South	76 (55.5%)	30 (61.2%)	46 (52.2%)
Number of Past tick bite(s)	1–2 bites	25 (18.2%)	9 (18.4%)	16 (18.2%)
	3–4 bites	2 (1.4%)	1 (2.0%)	1 (1.1%)
	5 + bites	5 (3.6%)	2 (4.1%)	3 (3.4%)
	Unsure	30 (21.9%)	11 (22.4%)	19 (21.6%)
	Never	69 (50.4%)	23 (46.9%)	46 (52.2%)

Note: Due to missing data, for some variables, the category totals do not add up to 137.

**Table 2:** Anticipated early symptoms of Lyme disease.

Symptoms	*n* = 79 (%)
Rash/Bull’s Eye Rash	16 (11.7)
Flu	11 (8.0)
Rash and Flu	10 (7.3)
Rash, Flu and Pain	3 (2.2)
Rash/Bull’s Eye Rash and Flu	49 (35.8)

## Supplementary Materials

The following are available online at https://www.mdpi.com/1660-4601/17/1/355/s1, Supplementary Materials: Knowledge of Tick Exposure and Lyme Disease.

## References

[1] Boudreau C.R., Lloyd V.K., Gould O.N. (2018). Motivation and experiences of Canadians seeking treatment for Lyme disease outside of the conventional Canadian health-care system. J. Patient Exp..

[2] Ogden N.H., Lindsay L.R., Morshed M., Sockett P.N., Artsob H. (2009). The emergence of Lyme disease in Canada. CMAJ.

[3] Government of Canada (2018). Risk of Lyme Disease to Canadians. https://www.canada.ca/en/public-health/services/diseases/lyme-disease/risk-lyme-disease.html.

[4] Hildenbrand P., Craven D.E., Jones R., Nemeskal P. (2009). Lyme neuroborreliosis: Manifestations of a rapidly emerging zoonosis. Am. J. Neuroradiol..

[5] Lloyd V.K., Hawkins R.G. (2018). Under-detection of Lyme disease in Canada. Healthcare.

[6] Ogden N.H., Arsenault J., Hatchette T.F., Mechai S., Lindsay L.R. (2017). Antibody responses to *Borrelia burgdorferi* detected by western blot vary geographically in Canada. PLoS ONE.

[7] Willhemsson P., Fryland L., Lindblom P., Sjowall J., Ahlm C., Berglund J., Haglund M., Henningsson A.J., Nolskog P., Nordberg M. (2016). A prospective study on the incidence of Borrelia burgdorferi sensu lato infection after a tick bite in Sweden and on the Åland Islands Finland (2008–2009). Ticks Tick Borne Dis..

[8] Canadian Lyme Disease Foundations (n.d.). https://canlyme.com/lyme-prevention/.

[9] Canada Centre for Occupational Health and Safety (2016). Lyme disease: A warm weather hazard. Health Saf. Rep..

[10] Cisak E., Zajac V., Wojcik-Fatla A., Dutkiewicz J. (2012). Risk of tick-borne diseases in various categories of employment among forestry workers in eastern Poland. Ann. Agric. Environ. Med..

[11] Kurnatowski P., Warpechowska M., Kurnatowska A.J. (2011). Knowledge on Lyme disease among foresters. Int. J. Occup. Med. Environ. Health.

[12] Thorin C., Rigaud E., Capek I., André-Fontaine G., Oster B., Gastinger G., Abadia G. (2008). Séroprévalence de la borréliose de Lyme et de l’encéphalite à tiques chez des professionnels exposés dans le Grand Est de la France. Med. Mal. Infect..

[13] Valente S.L., Wemple D., Ramos S., Cashman S.B., Savageau J.A. (2015). Preventive behaviors and knowledge of tick-borne illnesses: Results of a survey from an endemic area. J. Public Health Manag. Pract..

[14] Zajac V., Pinkas J., Wojcik-Fatla A., Dutkiewicz J., Owoc A., Bojar I. (2016). Prevalence of serological response to borrelia burgdorferi in farmers from eastern and central Poland. Eur. J. Clin. Microbiol. Infect. Dis..

[15] Herrington J.E.J., Campbell G.L., Bailey R.E., Cartter M.L., Adams M., Frazier E.L., Damrow T.A., Gensheimer K.F. (1997). Predisposing factors for individuals’ Lyme disease prevention practices: Connecticut, Maine, and Montana. Am. J. Public Health.

[16] Aenishaenslin C., Bouchard C., Koffi J.K., Pelcat Y., Ogden N.H. (2016). Evidence of rapid changes in Lyme disease awareness in Canada. Ticks Tick Borne Dis..

[17] Bayles B.R., Evans G., Allan B.F. (2013). Knowledge and prevention of tick-borne diseases vary across an urban-to-rural human land-use gradient. Ticks Tick Borne Dis..

[18] Butler A.D., Sedghi T., Petrini J.R., Ahmadi R. (2015). Tick-borne disease preventative practices and perceptions in an endemic area. Ticks Tick Borne Dis..

[19] Aenishaenslin C., Michel P., Ravel A., Gern L., Milord F., Waaub J.P., Belanger D. (2015). Factors associated with preventive behaviors regarding Lyme disease in Canada and Switzerland: A comparative study. BMC Public Health.

[20] Hallman W., Weinstein N., Kadakia S., Chess C. (1995). Precautions taken against lyme disease at three recreational parks in endemic areas of New Jersey. Environ. Behav..

[21] Public Health Agency of Canada (2017). Lyme Disease in Canada: A Federal Framework. https://www.canada.ca/en/public-health/services/publications/diseases-conditions/lyme-disease-canada-federal-framework.html.

[22] Government of New Brunswick (Office of the Chief Medical Officer of Health): Tick-Borne Diseases. https://www2.gnb.ca/content/gnb/en/departments/ocmoh/cdc/content/vectorborne_andzoonotic/Tick-Borne_Diseases.html.

