# Supplementary material for: Knowledge and Knowledge Needs about Lyme Disease among Occupational and Recreational Users of the Outdoors
Source: Int J Environ Res Public Health. 2020 Jan 5;17(1):355. doi: 10.3390/ijerph17010355 (PMC6981950; doi:10.3390/ijerph17010355)
Supplement: Supplementary file 1 [file ijerph-17-00355-s001.pdf]

Knowledge of Tick Exposure and Lyme Disease

In this survey, we are asking you questions about your knowledge of ticks and Lyme disease in order to help plan future education initiatives. We very much appreciate your willingness to participate in this research project.

1) How old are you? ________

2) Which best describes your gender:

- ☐ Male
- ☐ Female
- ☐ Other or prefer not to answer

3) Which best describes your highest level of education?

- ☐ Post-secondary education (i.e.,: university, college, trade school)
- ☐ High school
- ☐ Junior High School
- ☐ Elementary School

4) Which of the following **outdoor** activities best describe you (**select all that apply**):

- ☐ Sport Hunting
- ☐ Sport Fishing
- ☐ Small wood lot owner (logging)
- ☐ Farming
- ☐ Home Gardening
- ☐ Dog owner
- ☐ Recreational paddling (kayak, canoe, etc.)
- ☐ Tree Planting
- ☐ Recreational Hiking
- ☐ Golfing
- ☐ Horseback riding
- ☐ Other: ______________

5) In which county do you live?

- ☐ Albert
- ☐ Carleton
- ☐ Charlotte
- ☐ Gloucester
- ☐ Kent
- ☐ Kings
- ☐ Madawaska
- ☐ Northumberland
- ☐ Queens
- ☐ Restigouche
- ☐ Saint John
- ☐ Sunbury
- ☐ Victoria

- ☐ Westmorland
- ☐ York

6) How would you describe where you live?

- ☐ Urban (in a city)
- ☐ Suburban (near a city/small town)
- ☐ Rural

7) Please rate your own level of knowledge surrounding Lyme disease:
- ☐ Very little knowledge
- ☐
- ☐
- ☐
- ☐
- ☐ Very high level of knowledge

8) Have you ever had Lyme disease?

- ☐ Yes
- ☐ No
- ☐ Unsure

9) If **yes**, were you:
- ☐ Diagnosed by a health care provider
- ☐ Self-diagnosed

10) If you have had Lyme disease, when did your symptoms begin?

- ☐ Within the past 6 months
- ☐ Within the past 1-2 years
- ☐ Within 5 years
- ☐ 6 or more years ago

11) Are you still suffering from Lyme disease?

- ☐ Yes
- ☐ No

12) Please rate how frequently you use the following strategies to avoid ticks infected with Lyme disease
   a) How often do you wear long pants to avoid ticks infected with Lyme disease?
      - ☐ Never
      - ☐
      - ☐
      - ☐ Sometimes
      - ☐
      - ☐
      - ☐ Always

b) How often do you tuck your pants into your socks to avoid ticks infected with Lyme disease?
- ☐ Never
- ☐
- ☐
- ☐ Sometimes
- ☐
- ☐
- ☐ Always

c) How often do you wear boots or close-toed shoes to avoid ticks infected with Lyme disease?
- ☐ Never
- ☐
- ☐
- ☐ Sometimes
- ☐
- ☐
- ☐ Always

d) How often do you use a bug repellent spray on your body to avoid ticks infected with Lyme disease?
- ☐ Never
- ☐
- ☐
- ☐ Sometimes
- ☐
- ☐
- ☐ Always

e) How often do you use insecticide on the environment in your yard or work area to avoid ticks infected with Lyme disease?
- ☐ Never
- ☐
- ☐
- ☐ Sometimes
- ☐
- ☐
- ☐ Always

f) How often do you check your body for ticks to avoid ticks infected with Lyme disease?
- ☐ Never
- ☐
- ☐
- ☐ Sometimes
- ☐
- ☐
- ☐ Always

Formatted: Font: Palatino Linotype, 10 pt

g) How often do you avoid sitting in grassy areas to avoid ticks infected with Lyme disease?
- ☐ Never
- ☐
- ☐
- ☐ Sometimes
- ☐

- ☐
- ☐ Always

h) How often do you avoid walking in or near long grass to avoid ticks infected with Lyme disease?
- ☐ Never
- ☐
- ☐
- ☐ Sometimes
- ☐
- ☐
- ☐ Always

i) How often do you shower after you have been in a tick-infested area to avoid ticks infected with Lyme disease?
- ☐ Never
- ☐
- ☐
- ☐ Sometimes
- ☐
- ☐
- ☐ Always

13) Please rate the effectiveness of each of the following strategies in avoiding ticks infected with Lyme disease.

a) How effective do you think wearing long pants is in avoiding ticks infected with Lyme disease?
- ☐ Not at all effective
- ☐
- ☐
- ☐ Neutral
- ☐
- ☐
- ☐ Very Effective

b) How effective do you think tucking your pants into your socks is in avoiding ticks infected with Lyme disease?
- ☐ Not at all effective
- ☐
- ☐
- ☐ Neutral
- ☐
- ☐
- ☐ Very Effective

c) How effective do you think wearing protective footwear is in avoiding ticks infected with Lyme disease?
- ☐ Not at all effective
- ☐
- ☐
- ☐ Neutral
- ☐
- ☐
- ☐ Very Effective

How effective do you think using a bug repellent spray on your body is in avoiding ticks infected with Lyme disease?

- ☐ Not at all effective
- ☐
- ☐
- ☐ Neutral
- ☐
- ☐
- ☐ Very Effective

How effective do you think using insecticide on the environment in your yard or work area is in avoiding ticks infected with Lyme disease?

- ☐ Not at all effective
- ☐
- ☐
- ☐ Neutral
- ☐
- ☐
- ☐ Very Effective

d) How effective do you think checking your body for ticks is in avoiding ticks infected with Lyme disease?

- ☐ Not at all effective
- ☐
- ☐
- ☐ Neutral
- ☐
- ☐
- ☐ Very Effective

e) How effective do you think avoiding sitting in grassy areas is in avoiding ticks infected with Lyme disease?

- ☐ Not at all effective
- ☐
- ☐
- ☐ Neutral
- ☐
- ☐
- ☐ Very Effective

f) How effective do you think avoiding long grass is in avoiding ticks infected with Lyme disease?

- ☐ Not at all effective
- ☐
- ☐
- ☐ Neutral
- ☐
- ☐
- ☐ Very Effective

g) How effective do you think showering after you have been in a tick-infested area is in avoiding ticks infected with Lyme disease?

- ☐ Not at all effective
- ☐
- ☐
- ☐ Neutral

- ☐
- ☐
- ☐ Very Effective

14) Are there other things that you do to avoid contracting Lyme disease? Please explain.

____

15) Have you ever had a tick bite?

- ☐ Never
- ☐ 1-2 times
- ☐ 3- 4 times
- ☐ 5 or more times
- ☐ Unsure

16) If you have been bitten by a tick, how did you remove the tick?

- ☐ Used my fingers to pull out the tick
- ☐ Used tweezers to pull out the tick
- ☐ Used grease/gasoline, alcohol or other substance to kill the tick before I pulled it off
- ☐ Other (please explain): ____ __________________

17) If you removed a tick, did you:

- ☐ Get it tested
- ☐ Throw it away

18) When someone becomes infected with Lyme disease, what symptoms are they likely to have in the 1 to 4 weeks following the tick bite? **Please list ALL the symptoms that you think occur 1 to 4 weeks after becoming infected with Lyme disease.**

19) In your opinion, what is the best treatment for Lyme disease? **(select all that apply)**

- ☐ Nothing, it resolves over time
- ☐ Skin cream to control the rash
- ☐ Antibiotics prescribed by a physician
- ☐ Integrated care addressing many symptoms at the same time
- ☐ Other (Please explain): ____

____

____

20) Where have you learned about ticks and Lyme disease? (**select all that apply**)

- ☐ Training at work
- ☐ Health care provider (nurse and/or doctor)
- ☐ Public Health (Pamphlets, information sheets and websites prepared by government agencies)
- ☐ Educational programs from recreational association (hunting, fishing, etc.)
- ☐ News media (newspapers, magazines, TV news, or radio)
- ☐ Social media (Facebook and/or Twitter)
- ☐ Friends and/or family
- ☐ Other (please explain) ______________________

21) Where would you <u>like to learn more</u> about ticks and Lyme disease? (**select all that apply**)

- ☐ Training at work
- ☐ Health care provider (nurse and/or doctor)
- ☐ Public Health (Pamphlets and information sheets prepared by government agencies)
- ☐ Educational programs from recreational association (hunting, fishing, etc.)
- ☐ News media (newspapers, magazines, TV news, or radio)
- ☐ Social media (Facebook and/or Twitter)
- ☐ Friends and/or family
- ☐ Other (please explain) ______________________

22) What would you like to know more about concerning Lyme disease?

a) How to prevent a tick bite

- ☐ Not at all important for me to learn more about this
- ☐
- ☐
- ☐ Neutral
- ☐
- ☐
- ☐ Very important for me to learn about this

b) How to deal with tick bites

- ☐ Not at all important for me to learn more about this
- ☐
- ☐
- ☐ Neutral
- ☐
- ☐
- ☐ Very important for me to learn about this

c) How to recognize Lyme disease in the first days of infection

- ☐ Not at all important for me to learn more about this
- ☐
- ☐
- ☐ Neutral
- ☐
- ☐

- ☐ Very important for me to learn about this

d) How to recognize long-term effects of Lyme disease

- ☐ Not at all important for me to learn more about this
- ☐
- ☐
- ☐ Neutral
- ☐
- ☐
- ☐ Very important for me to learn about this

e) What are the treatments for Lyme disease

- ☐ Not at all important for me to learn more about this
- ☐
- ☐
- ☐ Neutral
- ☐
- ☐
- ☐ Very important for me to learn about this

f) Where do I get treatment for Lyme disease?

- ☐ Not at all important for me to learn more about this
- ☐
- ☐
- ☐ Neutral
- ☐
- ☐
- ☐ Very important for me to learn about this

g) How to identify ticks versus other insects or bugs

- ☐ Not at all important for me to learn more about this
- ☐
- ☐
- ☐ Neutral
- ☐
- ☐
- ☐ Very important for me to learn about this

h) Where to get a tick tested to find out if it carries Lyme disease

- ☐ Not at all important for me to learn more about this
- ☐
- ☐
- ☐ Neutral
- ☐
- ☐
- ☐ Very important for me to learn about this

Formatted: Font: Palatino Linotype, 10 pt

i) Where to find health care providers who are knowledgeable about Lyme disease

- ☐ Not at all important for me to learn more about this
- ☐
- ☐
- ☐ Neutral
- ☐
- ☐
- ☐ Very important for me to learn about this

j) How to find out if an area contains many ticks infected with Lyme disease (endemic)

- ☐ Not at all important for me to learn more about this
- ☐
- ☐
- ☐ Neutral
- ☐
- ☐
- ☐ Very important for me to learn about this

23) What are other aspects of Lyme disease that you would like more information about? Please explain.

____

This is the end of our questions. Please use the space below for any additional comments regarding Lyme disease that you would like to share with us.

____

We very much appreciate your help with this survey.
